# Minoxidil-Coated Lysozyme-Shelled Microbubbes Combined With Ultrasound for the Enhancement of Hair Follicle Growth: Efficacy *In Vitro* and *In Vivo*

**DOI:** 10.3389/fphar.2021.668754

**Published:** 2021-04-27

**Authors:** Ai-Ho Liao, Yu-Jhen Huang, Ho-Chiao Chuang, Chih-Hung Wang, Cheng-Ping Shih, Chien-Ping Chiang

**Affiliations:** ^1^ Graduate Institute of Biomedical Engineering, National Taiwan University of Science and Technology, Taipei, Taiwan; ^2^ Department of Biomedical Engineering, National Defense Medical Center, Taipei, Taiwan; ^3^ Department of Mechanical Engineering, National Taipei University of Technology, Taipei, Taiwan; ^4^ Graduate Institute of Medical Sciences, National Defense Medical Center, Taipei, Taiwan; ^5^ Department of Otolaryngology–Head and Neck Surgery, Tri-Service General Hospital, National Defense Medical Center, Taipei, Taiwan; ^6^ Taichung Armed Forces General Hospital, Taichung, Taiwan; ^7^ Department of Dermatology, Tri-Service General Hospital, National Defense Medical Center, Taipei, Taiwan; ^8^ Department of Biochemistry, National Defense Medical Center, Taipei, Taiwan

**Keywords:** ultrasound, cavitation, lysozyme-shelled microbubbles, minoxidil, hair follicle

## Abstract

Lysozyme (Lyz) is an antimicrobial peptide, a safe adjunct, and it has been indicated that Lyz can promote vibrissae follicle growth by enhancing the hair-inductive capacity of dermal papilla cells in mice. The present study produced a new type of minoxidil (Mx)-coated antifungal Lyz-shelled microbubble (LyzMB) for inhibiting bacteria and allergies on the oily scalp. The potential of Mx-coated LyzMBs (Mx-LyzMBs) combined with ultrasound (US) and the role of LyzMB fragments in enhancing hair follicle growth were investigated. Mx grafted with LyzMBs were synthesized and the loading efficiency of Mx on cationic LyzMBs was 20.3%. The biological activity of Lyz in skin was determined using an activity assay kit and immunohistochemistry expression, and the activities in the US+Mx-LyzMBs group were 65.8 and 118.5 μU/mL at 6 and 18 h, respectively. In hair follicle cell culture experiments, the lengths of hair follicle cells were significantly enhanced in the US+Mx-LyzMBs group (108.2 ± 11.6 μm) compared to in the US+LyzMBs+Mx group (44.3 ± 9.8 μm) and the group with Mx alone (79.6 ± 12.0 μm) on day 2 (*p* < 0.001). During 21 days of treatment in animal experiments, the growth rates at days 10 and 14 in the US+Mx-LyzMBs group increased by 19.4 and 65.7%, respectively, and there were significant differences (*p* < 0.05) between the US+Mx-LyzMBs group and the other four groups. These findings indicate that 1-MHz US (applied at 3 W/cm^2^, acoustic pressure = 0.266 MPa) for 1 min combined with Mx-LyzMBs can significantly increase more penetration of Mx and LyzMB fragments into skin and enhance hair growth than Mx alone.

## Highlights

- Combining lysozyme shelled microbubbles and minoxidil with ultrasound can promote hair growth enhancements more significant than minoxidil alone.
- Minoxidil can be grafted directly as a layer on lysozyme shelled microbubbles, which results in a higher loading efficiency than for albumin-shelled MBs.
- Ultrasound can enhance the delivery of lysozyme shelled microbubbles fragments closer to the target structure of interest in the hair follicles.
- A microbubbles-based drug carrier system for minoxidil could be developed using lysozyme shelled microbubbles.

## Introduction

Topical minoxidil (Mx; Rogaine, Johnson & Johnson Consumer Inc., Skillman, NJ, United States) is the only medication that can be used by both men and women and approved by the United States Food and Drug Administration for the treatment of androgenetic alopecia (Springer et al., 2003). It does not work on completely bald areas and only works over the long term if it is used continuously (Springer et al., 2003). Infection and microinflammation of the hair follicles have been considered to affect the pathogenesis of androgenic alopecia even during treatment with Mx (Sakr et al., 2013). Piérard et al. found that applying topical antimicrobials may be beneficial for the treatment of androgenic alopecia (Piérard et al., 1996). Compared with using Mx alone, combination agents containing Mx-pyrithione zinc (topical antimicrobial agent), Mx-ketoconazole (topical antifungal agent), and Mx-hydrocortisone (topical anti-inflammatory steroid) have been shown to improve hair growth (Kligman, 1991; Khandpour et al., 2002; Berger et al., 2003). Our previous study has provided a new integrated transdermal drug delivery platform for monitoring the delivery of Mx to hair follicles by utilizing multifunctional albumin-shelled microbubbles (MBs) and ultrasound (US) (Liao et al., 2016b). The present study investigated the potential of Mx-coated antifungal lysozyme (Lyz)-shelled MBs (Mx-LyzMBs) combined with US and the role of Lyz-shelled MB (LyzMB) fragments in enhancing hair follicle growth.

Three genes (lysozyme c, lactalbumin, and calcium-binding lysozyme) have been considered to compose the traditional vertebrate Lyz gene family (Irwin et al., 2011). Chicken-type Lyz c (found at high concentrations in the eggs of many bird species) is a bacteriolytic enzyme that is secreted into many body fluids of mammals, such as blood, tears, and breast milk (McKenzie and White Jr, 1991; Callewaert and Michiels, 2010). It has been suggested that the Lyz gene family is associated with the development of new hairs in mammals (Irwin et al., 2011). Lyz also has an antimicrobial property and acts as a cell-wall lytic enzyme that degrades murein in the cell wall via the cleavage of the *β* glycosidic bond between N-acetylmuramic acid and N-acetylglucosamine (Su et al., 2015). It was suggested that this could influence skin inflammation, modify wound healing, provide greater insight into the pathophysiology of skin disorders, and offer new therapeutic opportunities (Yamasaki and Gallo, 2008). In 2015, a study revealed that Lyz can stimulate the growth of mouse hair follicles *in vitro* (Su et al., 2015). More information about the exact location of Lyz in hair follicles and whether it exerts any direct effects on hair follicle growth should therefore be investigated. Moreover, Lyz is a biochemical defense agent secreted by the skin and also found at relatively high concentrations on the skin surface. A previous study suggested that the human epidermis has the ability to synthesize Lyz and provide antibacterial protection (Chen et al., 1986).

Our previous study used Lyz as the shell of MBs and combined MBs with US to reduce the dose and treatment duration and improve the prognosis of acne vulgaris (Liao et al., 2017). We have also integrated epidermal growth-factor-coated LyzMBs into a wound dressing and combined this with US as a new platform for enhancing the healing and prognosis of a wounded area both *in vitro* and *in vivo* (Liao et al., 2018).

Small gas-filled colloidal MBs are commonly applied in clinical applications as contrast agents for US imaging via intravenous injection. The shell of such MBs is primarily based on protein, polymer, or lipid coatings. MBs have recently been approved for use in a large diversity of contrast-enhanced US, molecular imaging, and US-mediated drug delivery applications (Di Ianni et al., 2019; Hernández-Gil et al., 2019; Yang et al., 2019). Our previous studies have demonstrated the effects of using different conditions of albumin-shelled MBs for enhancing their penetration in transdermal delivery *in vivo* (Liao et al., 2015; Liao et al., 2016a; Liao et al., 2016c). Layer-by-layer albumin-shelled MBs that absorb chitosan oligosaccharide lactate (COL) and Mx (Mx-COL-MBs) were created, and they were combined with energy from sonication by US in the water phase to enhance hair growth while shortening the treatment period (Liao et al., 2016b).

In these previous studies the MBs played the role of an enhancer and did not direct treat the skin layer, even when it was disrupted into fragments by the US. The present study investigated the role of LyzMB fragments combined with US and Mx for enhancing hair follicle growth both *in vitro* and *in vivo*. The amounts of Mx and Lyz that after 18 h had permeated the skin, deposited on the skin, and penetrated the skin were evaluated.

## Materials and Methods

### Preparation and Characterization of Mx-Coated LyzMBs

**FIGURE 1:**
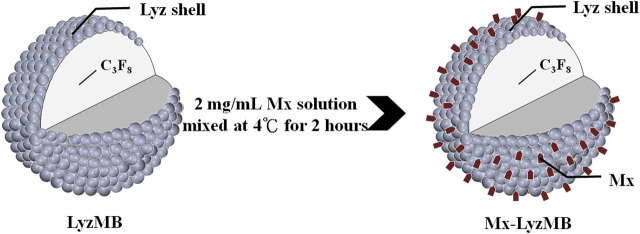
Schematics (not to scale) of a LyzMB and an Mx-coated LyzMB (Mx in red color).

The composition of the prepared self-assembled Mx-LyzMBs is presented in Figure 1 and Table 1. Since Lyz has a positive charge, the surface potential of the Lyz shell exceeds zero, and so it can attract molecules with negative charges. Therefore, the Lyz shell with positive charges can be adsorbed onto Mx by electrical adsorption since Mx contains negatively charged oxygen atoms. This results in Mx being distributed over the outside shell surface of Lyz, and produces Mx-LyzMBs through the modification by Mx. LyzMBs were prepared according to the procedure used in our previous study (Liao et al., 2017; Liao et al., 2018). In brief, 50 mg of chicken-egg-white Lyz was dissolved in 1 ml of 50 mM Tris buffer (pH 8), 20 mg of reducing agent (DL-Dithiothreitol) was added, and the solution was then shaken at 50 rpm for 15 min at room temperature to allow sufficient time for partial reduction to occur. LyzMBs were generated by sonicating this solution in perfluoropropane gas using a sonicator at a power of 120 W (Branson Ultrasonics, Danbury, CT, United States) for 30 s. The LyzMBs were centrifuged at 1,200 rpm (128.6×*g*) for 2 min and then washed three times to eliminate the Tris buffer and DL-DTT using Milli-Q water (pH = 6.4, resistance = 18.2 mΩ). Before incubation with Mx (molecular weight = 209.25; Sigma-Aldrich, St. Louis, IL, United States), the LyzMBs were centrifuged (1,200 rpm, 128.7×*g*; F2402 rotor, Beckman Coulter, Fullerton, CA, United States) for 1 min and then the Milli-Q water was removed. Mx (2 mg/ml) was then incubated with the produced LyzMBs on a rotary shaker (15 rpm; Shaker RS-01, TKS, New Taipei City, Taiwan) for 2 h at 4°C in a refrigerator to produce Mx-LyzMBs. These Mx-LyzMBs were washed three times to remove the unbound Mx. The number of Mx-LyzMBs in the solution was measured using the MultiSizer III device (Beckman Coulter) with a 30-μm aperture probe whose measurement boundary ranged from 0.6 to 20 μm. The size distribution in the suspension was measured using dynamic light scattering (Nanoparticle Analyzer, Horiba, Kyoto, Japan).

**GRAPHICAL ABSTRACT:**
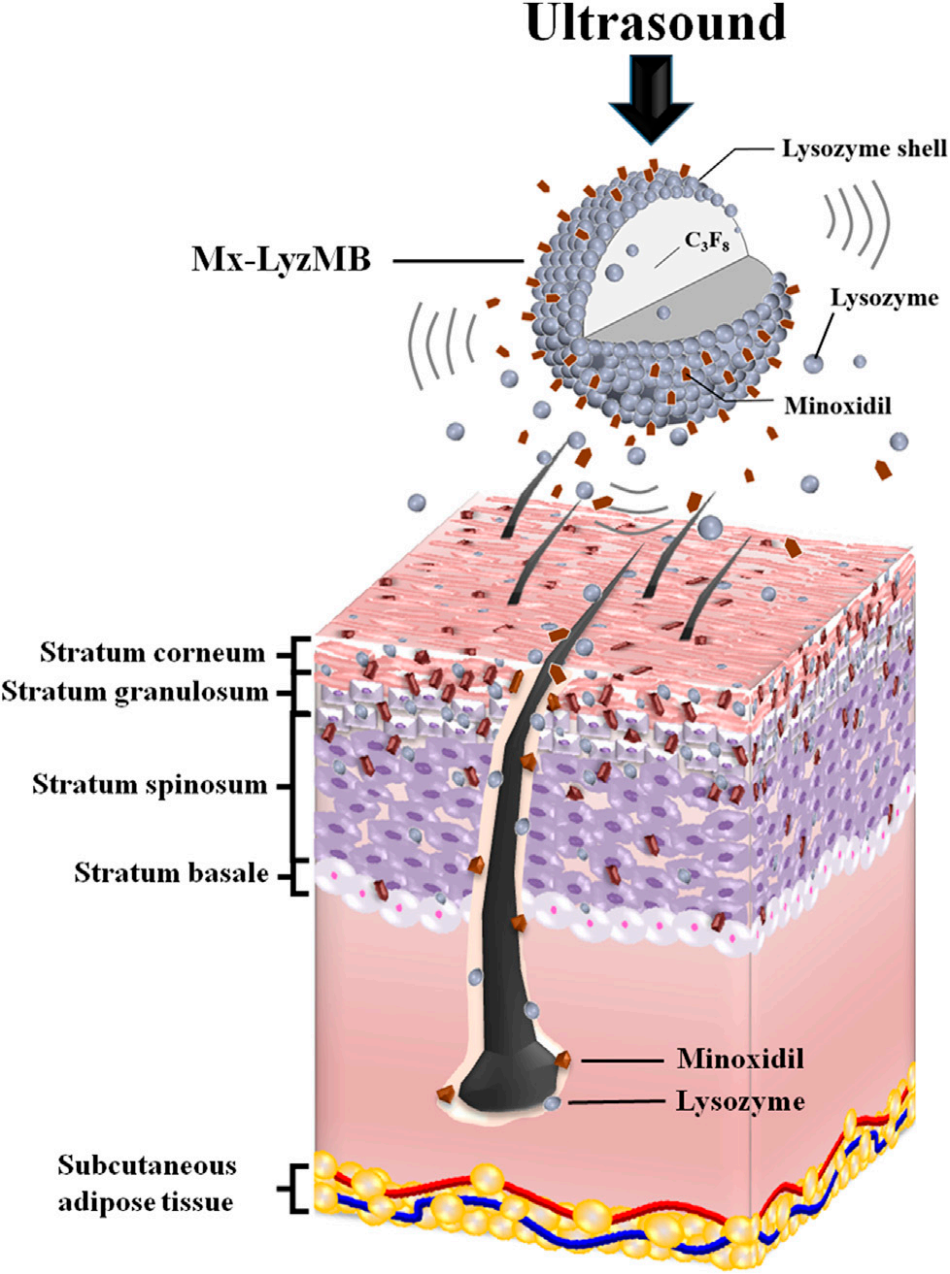
US can deliver Mx and LyzMB fragments closer to the target structure of interest in the hair follicles and enhance the amounts of Mx and Lyz either deposited on the skin or penetrating the skin. Mx release was more rapid when combining US with Mx-LyzMBs than with LyzMBs+Mx.

**TABLE 1:** Composition and Mx encapsulation efficiency of various LyzMBs.

Parameters	Mx:Lyz	Mx (mg/ml)	Encapsulation efficiency (%)
Groups			
LyzMBs	N/A	N/A	N/A
Mx solution	N/A	2	N/A
Mx-LyzMBs (0.5:1)	0.5:1	0.5	14.31 ± 5.95
Mx-LyzMBs (1:1)	1:1	1	16.70 ± 1.53
Mx-LyzMBs (2:1)	2:1	2	20.33 ± 2.37

The morphology of the Mx-LyzMBs was characterized by filtering 40-fold-diluted Mx-LyzMBs using a 5-μm syringe filter (Sartorius, Goettingen, Germany), and then 5 μL of the Mx-LyzMBs was studied using scanning electron microscopy (SEM) after coating the samples with platinum at 20 mA for 20 min using an automatic sputter coater (JFC-1300, JEOL, Tokyo, Japan). SEM images were recorded at an accelerating voltage of 15 kV.

### *In Vitro* Release Study

The *in vitro* release and penetration depth in pigskin were investigated according to the procedures used in our previous study (Liao et al., 2016b; Liao et al., 2018). In brief, in the *in vitro* release study, 3 ml (2.33×10^7^ bubbles/mL) of an Mx-LyzMB suspension (of the original concentration after production) was loaded into a dialysis bag (molecular weight cutoff = 12,000–14,000) and dialyzed against the release media of phosphate-buffered saline (PBS) at pH values of 5.0 and 7.4 and at 36.5–37.5°C, with stirring by a magnetic bar at 600 rpm. After 30 min, the 1-MHz unfocused US transducer (diameter 12 mm) of a sonoporation system (ST 2000V, NepaGene, Ichikawa, Japan) positioned 3 mm from the top of the dialysis bag under the liquid level provided sonication at a power density of 3 W/cm^2^ [acoustic pressure = 0.266 MPa, with a duty cycle of 50% and a pulse repetition period (PRP) of 250 ms] for 5 min. One-milliliter samples were taken from the release medium after times of 0.06, 0.12, 0.18, 0.24, 0.30, 1, 2, 3, 4, 5, and 6 h, with the same volume of PBS added each time to replace the release medium. These samples were analyzed using a UV-visual spectrophotometer (Lambda 40, PerkinElmer, Bridgeville, PA, United States). The drug release profile of Mx was examined as a control.

### *In Vitro* Skin Penetration by Mx and Lyz

Samples of fresh porcine ear skin with hair follicles were obtained from the Affiliated Slaughterhouse of New Taipei City Meat Market, and all experiments involving them were completed within 6 h. A 1-mm-thick sample of pigskin was harvested using a Humby knife, carefully cleaned with PBS, and cut into square pieces (2 cm × 2 cm). The *in vitro* skin penetration was then tested using static Franz diffusion cells over an area of 2.14 cm^2^ according to the experimental design used in our previous study (Liao et al., 2016c). The temperature of the diffusion assembly was maintained at 37°C. The probe of the sonoporation system, LyzMBs, 0.4 mg of Mx (molecular weight = 209.25, Sigma-Aldrich), or Mx-LyzMBs (containing 0.4 mg of Mx) in PBS (1 ml) (as a control) were applied to the donor cells facing the stratum corneum side of the skin, and occluded with Parafilm (Pechiney Laboratory Safety Products and Apparel, Chicago, IL, United States). The receptor diffusion half cell facing the dermis side was filled with PBS (pH 7.4, 4.5 ml); that cell contained a magnetic stirring bar rotating at 600 rpm as well as 0.01% gentamicin to prevent bacterial degradation of the Mx during the penetration process. Solutions in the diffusion cell without MBs were filtered through a 0.2-μm micropore filter (Nalgene, Rochester, NY, United States) or a 0.22-μm micropore filter (Millex, Darmstadt, Germany). Aliquots (200 μL) of receptor solution were taken after various times (0.58, 1, 2, 3, 4, 5, 6, 8, 12, and 18 h), with the cell refilled each time with the same volume of fresh receptor solution. After 30 min, the 1-MHz US transducer of the sonoporation system (ST 2000V, NepaGene) positioned 3 mm from the top of the skin provided sonication at a power density of 3 W/cm^2^ (acoustic pressure = 0.266 MPa) for 1 min. Samples were kept in a freezer until being analyzed using a UV-visual spectrophotometer (Lambda 40, PerkinElmer).

At the end of the penetration experiments (i.e., after 18 h), the skin sample was detached from the diffusion cell and carefully rinsed five times with distilled water to remove excess Mx from its surface. The skin was cut into 0.1-g pieces and homogenized with 1 ml of receptor solution for 2 min at 10,000 rpm (Polytron-Aggregate PT3100, Kinematica, Lucerne, Switzerland). The homogenized suspension was centrifuged for 25 min at 3,100×*g* (Thermo Fisher Scientific, Bremen, Germany), and then the concentrations of Mx and Lyz in the supernatant were determined using a UV-visual spectrophotometer (Lambda 40, PerkinElmer) and an enzyme-linked immunosorbent assay reader (Epoch, Biotek, Winooski, VT, United States), respectively. For the Mx measurements, sample volumes of 200 μL were added to the cuvette and placed in the spectrophotometer. For the Lyz measurements, samples were analyzed using the Lyz Activity Assay Kit (K236-100, BioVision, Francisco, CA, United States). The Mx or Lyz calibration curve served as the standard curve against which the absorption peaks and the corresponding concentrations of Mx or Lyz in the samples were measured.

### Measurement of Penetration Depth in Pigskin

The penetration depth in pigskin was measured by sonicating the treatment area of the sample by the 1-MHz US transducer of the sonoporation system attached to the top of the sample under the liquid level, and was performed successively at a power density of 3 W/cm^2^ (I_SPTA_ = 0.655 W/cm^2^) for 1 min after adding 500 μL of the LyzMBs with 0.1 mg of the model drug fluorescein isothiocyanate (FITC). The LyzMBs and FITC solution were left for 6 h after sonication, and after their removal the area was washed three times for 1 min each with PBS. The treated areas of pigskin were then embedded in an optimal-cutting-temperature solution (Surgipath FSC 22, Leica Microsystems, Buffalo Grove, IL, United States) on round specimen disks with a diameter of 2.2 cm. The distribution of the FITC in the cryosections was determined with the aid of an upright fluorescence microscope in the transmission and fluorescence modes (DM 2500, Leica Microsystems, Buffalo Grove, IL, United States) (Mak et al., 2012).

The chicken Lyz (the material source of LyzMBs) antigens were detected using an indirect enzyme immunohistochemistry method. Briefly, deparaffinized sections were immersed in absolute acetone for 30 min, and 0.5% hydrogen peroxide for 10 min to remove endogenous peroxidase activity. The sections were then rinsed three times with 0.05%-Tween-added 0.01 M PBS (TPBS; pH 7.4) after all preparation steps had been completed in order to remove any residue reagent. Following blocking with Universal Blocking Reagent 10X (BioGenex, Fremont, CA, United States) for 10 min at room temperature, the sections were reacted with anti-Lyz rabbit IgG (LS-C153796, LifeSpan BioSciences, Seattle, WA, United States; diluted at 1:500) for 1 h at 6°C. The super enhancer reagent was then added for Poly-HRP enhancement, and the sections were incubated with Poly-HRP reagent (BioGenex) for 30 min at room temperature. After rinsing with TPBS, the sections were incubated with DAB (3,3′-diaminobenzidine, Sigma-Aldrich) containing 0.03% H_2_O_2_ and counterstained with hematoxylin. Control sections were incubated with TPBS or nonimmunized rabbit IgG instead of the primary antibodies.

### Optimization of MBs Concentration for US-Mediated MB Destruction

**FIGURE 2:**
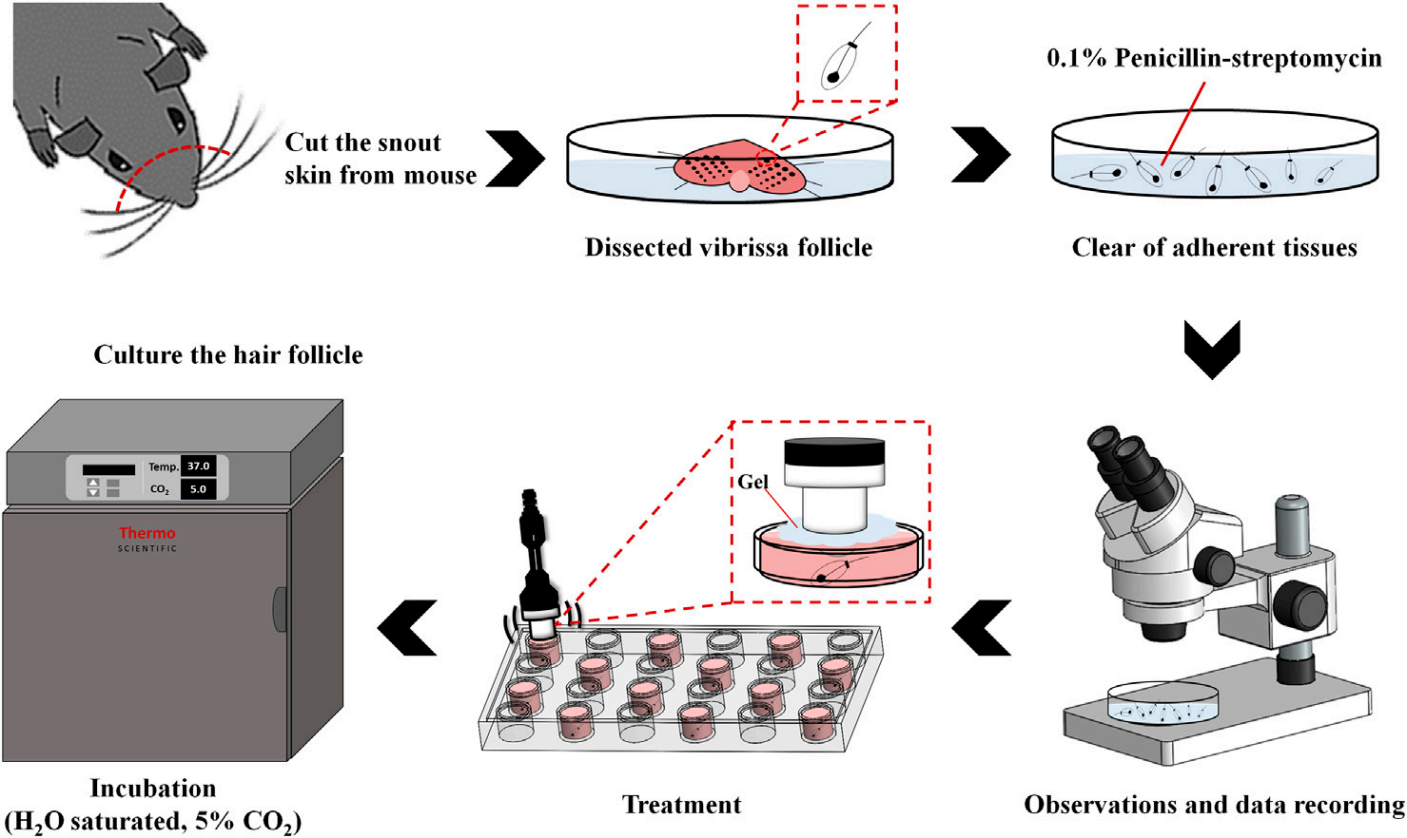
Schematic of the process of culturing *in vitro* murine vibrissae in serum-free medium.

Since the drug delivery enhancement is related to the destruction efficacy of MBs (Liao et al., 2020a; Liao et al., 2020b). As illustrated in Figure 2, each well of a 24-well plate was filled with about 4 ml of MBs (1.4 × 10^7^, 0.7 × 10^7^, or 0.35 × 10^7^ MBs/ml) while ensuring that no air bubbles were trapped when the cover was placed. A US instrument equipped with a 10-mm diameter probe operating at a center frequency of 1 MHz and duty cycle of 50% was used for sonication. The probe was placed directly on the 24-well cover, and gel was used as an ultrasound coupling agent. The US parameters for MB destruction *in vitro* were established by investigating the effects of US exposure at 1 W/cm^2^ for 30 s once, twice, three times, four times, or five times. After each US exposure, the MB solution in each well was diluted 10 times and then imaged using a US animal imaging system (Prospect, S-Sharp Corporation, Taipei, Taiwan). Images were processed with custom MATLAB programs (The MathWorks, Natick, MA, United States) to evaluate the destruction efficiency by calculating the difference in the gradient strength on the MB images between before and after US exposure. The *in vitro* effects of US-mediated MB destruction on hair follicle cells were evaluated by placing one cell in each well of a 24-well plate overnight. The next day each well was filled with about 4 ml of MBs (1.4 × 10^7^ MBs/ml), followed by US exposure as described above. After the US exposure, the MB solution was replaced with culture medium and the cells were allowed to grow for 48 h. The hair growth rate was observed every 2 days.

### Murine Vibrissae Cultured in Serum-Free Medium

Figure 2 shows a schematic diagram of the *in vitro* vibrissae culture procedure (Thuangtong et al., 2013). The anagen follicles were harvested from the upper lip of six-week-old C57BL/6J mice and placed in PBS (containing 0.1% penicillin-streptomycin) on ice. The hair follicle is epithelial except for a loose surrounding sheath of connective tissue and the dermal papilla. Only anagen follicles were selected (as identified by their characteristic morphology of melanized epithelial bulbs) and cut transversely about 3 mm from the base. Each isolated follicle was immediately placed into a well of a 24-well plate filled with 3 ml of culture medium, which consisted of 0.5 ml of William’s Medium E 1X (GlutaMAX™, Gibco, Paisley, Scotland, United Kingdom), L-glutamine (2 mM, Sigma-Aldrich), hydrocortisone (10 ng/ml, Gibco), insulin (10 μg/ml, Gibco), penicillin (100 U/ml, Gibco), and streptomycin (100 μg/ml, Gibco). All cultures were incubated at 37°C in an atmosphere of 5% CO_2_ and 95% air, and then divided into the following seven groups (*n* = 5 per group) for incubation with different combinations of US, LyzMBs, and Mx-LyzMBs with or without US exposure (1 W/cm^2^ for 30 s, four times): 1) control, 2) Lyz only (25 mg/ml), 3) Mx only (0.07 mg/ml), 4) US only, 5) LyzMBs (7 × 10^6^ MBs/ml) with US, 6) LyzMBs (7 × 10^6^ MBs/ml) mixed Mx with US, and 7) Mx-LyzMBs (7 × 10^6^ MBs/ml) with US. The treatments were performed and the medium was replaced every 2 days.

### Animal Treatments

Six-week-old C57BL/6J mice weighing 20–25 g were obtained from Bio Lasco (Taipei, Taiwan). The experimental protocol was approved by the Institutional Animal Care and Use Committee of the National Defense Medical Center, Taipei, Taiwan. Animals were cared for in compliance with institutional guidelines and regulations. Throughout the experiments, the animals were housed in stainless-steel cages in an air-conditioned room with the temperature maintained at 25–28°C and with alternating light and dark periods of 12 h each. The animals were acclimatized for 7 days prior to the experiments.

An area of about 10 cm^2^ on the dorsal skin of each animal was shaved using an animal clipper when the animal was 8 weeks of age, at which time all of the hair follicles were synchronized in the telogen phase. The skin color was measured using the CR-400 Chroma Meter device (Konica Minolta Sensing, Tokyo, Japan). The animals were divided into the following five groups (*n* = 6 per group, treatment applied once daily for 3 weeks): 1) no treatment (control), 2) US alone (US group), 3) US combined with LyzMBs (US+LyzMBs group), 4) US combined with LyzMBs and penetrating Mx (US+LyzMBs+Mx group), and 5) US combined with Mx-LyzMBs (US+Mx-LyzMBs group). The treated area in dorsal skin was a round area surrounded by a ring holder with a diameter of 2 cm. The 1-MHz US transducer was applied at 3 W/cm^2^ (acoustic pressure = 0.266 MPa) for 1 min, and 0.4 mg/ml (0.5 ml/cm^2^) Mx was used in all cases. The change in skin color induced by each of the treatments was assessed at predetermined times using the Chroma Meter device. The luminosity index, *L*, was calculated on each measurement day before and after treatment. The hair growth rate was calculated from *L* values using the following equation:

$$\mathrm{Hair}\;\mathrm{growth}\;\mathrm{rate}\left(\%\right)\;=\;\left[\frac{\left(L_{1}-L_{n}\right)}{L_{1}}\right]\times 100\%$$

where *L*_1_ is the luminosity index immediately after removing the hair and *L*_*n*_ is the luminosity index at each measurement time point.

### Histochemistry

Skin tissue samples (approximately 8 mm × 8 mm) were cut from the treatment area immediately after the experiments and then stored in a 10% formalin solution. Hematoxylin and eosin (Sigma-Aldrich) staining was applied, and the thickness, diameter, and number of hair follicles were analyzed using an image analysis system (TissueFAXS 3.5, TissueGnostics, Vienna, Austria) with the scanner (TissueQuest) and cytometry (HistoQuest) analysis packages provided with the system.

### Statistical Analysis

The obtained data were analyzed statistically using Student’s *t*-test for comparisons between two groups. Multiple groups were compared using one-way ANOVA followed by Bonferroni correction for multiple-comparisons test. Microsoft Office Excel 2016 and SigmaPlot 12.5 (Systat Software Inc., United States) were used for all statistical analysis. A probability value of *p* < 0.05 was considered indicative of a significant difference. Data are presented as mean ± SEM values.

## Results

### Characterization of Synthesized Mx-LyzMBs

**FIGURE 3:**
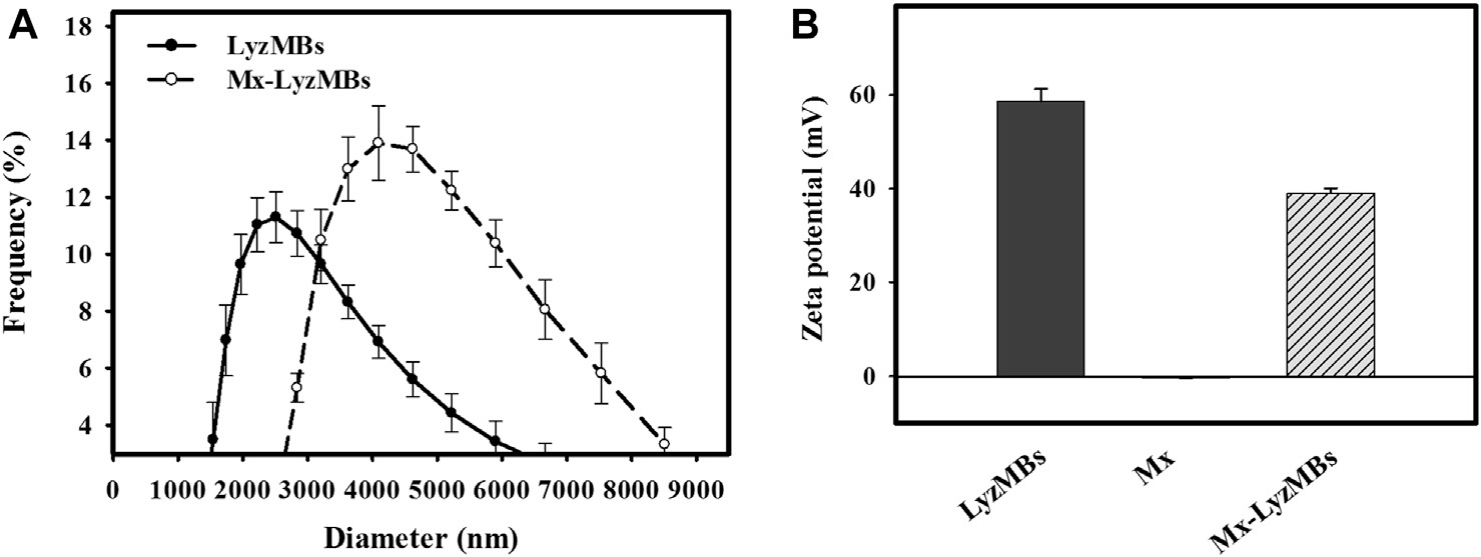
Quantification of the size distributions **(A)** and zeta potentials **(B)** of LyzMBs, Mx, and Mx-LyzMBs. Data are mean and SEM values.

The diameters of the LyzMBs and Mx-LyzMBs were 2.40 ± 0.14 and 4.29 ± 0.70 μm, respectively (Figure 3A); the corresponding concentration of LyzMBs was 4.67 ± 1.26×10^8^/ml (*n* = 7). The Lyz content in the original LyzMB solution constructed using the sonicator at a power of 120 W was 25 mg/ml. The zeta potentials of the LyzMBs, Mx, and Mx-LyzMBs dispersed in an aqueous solution (pH = 6.4, resistance = 18.2 mΩ) were measured using a Nanoparticle Analyzer (Horiba). Lyz is a positively charged protein at pH 7, and the LyzMBs had a potential of +58.60 ± 10.27 mV. The original potential of Mx (–0.36 ± 0.13 mV) was reversed to a surface potential of Mx-LyzMBs of +39.2 ± 1.09 mV (Figure 3B) (*n* = 9).

**FIGURE 4:**
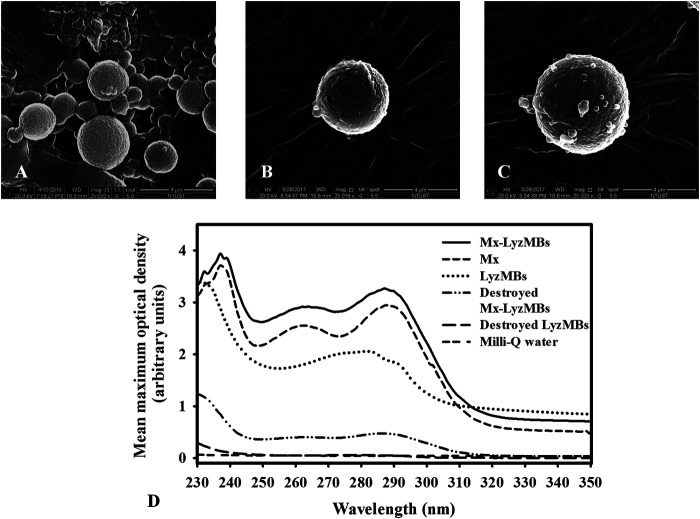
SEM images of **(A)** multiple LyzMBs, **(B)** a single LyzMB, and **(C)** a single Mx-LyzMB. **(D)** Absorbance spectra of Mx-LyzMBs, Mx, LyzMBs, Mx-LyzMBs after US exposure (destruction), LyzMBs after US exposure (destruction), and Milli-Q water.

Analysis of the encapsulation efficiency of the Mx coated on the LyzMB shells revealed a maximum loading efficiency of Mx-LyzMBs (with Mx:Lyz = 1:2) of 20.33 ± 2.37% in Table 1. These Mx-LyzMBs were used for the subsequent experiments involving *in vitro* skin penetration, *ex vivo* cultured murine vibrissae, and *in vivo* animal treatments. Figures 4A–C show SEM images of LyzMBs, a single LyzMB, and a single Mx-LyzMB, respectively. The composite structures of the LyzMB shell were observable by SEM, which indicated the presence of nanoscale particles after coating with Mx. Figure 4D shows the absorbance spectra of the Mx-LyzMBs, Mx, LyzMBs, Mx-LyzMBs after US exposure (destruction), LyzMBs after US exposure (destruction), and Milli-Q water. The results show that Lyz and LyzMBs absorb light at 280 nm, whereas Mx and Mx-LyzMBs absorb light at 240, 265, and 288 nm.

### *In vitro* Mx Release From LyzMBs

**FIGURE 5:**
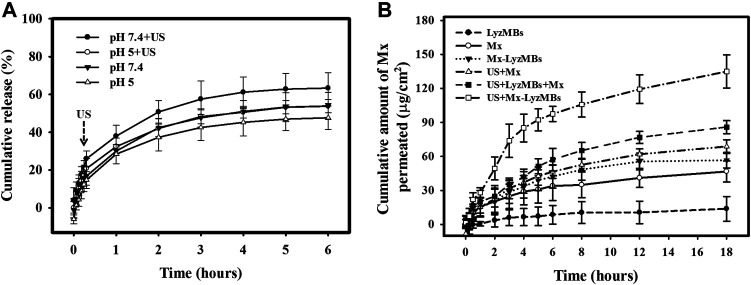
**(A)** Comparative cumulative drug release of Mx after 6 h from Mx-LyzMBs with and without US exposure in PBS at pH 5 and 7.4. **(B)** *In vitro* drug penetration in the different experimental groups (see Tables 1 , 2) through pigskin in a Franz diffusion cell at 36–37°C. Data are mean and SEM values.

Figure 5A shows the percentage cumulative Mx release after 6 h in the Mx-LyzMBs and US+Mx-LyzMBs groups in PBS for pH values of 5 and 7.4. At pH 5, 28.4% of the free Mx had diffused through the dialysis membrane relative to control over the first 1 h, and this increased to 32.4% with US exposure; at pH 7.4 these values were 30.0 and 38.0%, respectively. Without US exposure, the proportion of the free drug suspension that was released across the dialysis membrane was reduced to only 48.3% at pH 5 and 54.2% at pH 7.4 after 6 h. With US exposure, the *in vitro* release of the Mx was rapid during the first 3 h, reaching 48.3% at pH 5 and 57.5% at pH 7.4, followed by a slower but sustained release of Mx from the LyzMBs to just over 56.7% at pH 5 and 66.8% at pH 7.4 after 6 h. These findings indicate that the application of US energy enhanced drug release by 2.2–10.2%, and also that the pH value affects the efficiency of Mx release from LyzMBs.

### *In vitro* Skin Permeation of Mx From Mx-LyzMBs and Lyz From LyzMBs

Figure 5B shows the Mx concentrations in the six groups for percutaneous penetration over 18 h as analyzed using the UV-visual spectrophotometer. The concentration in all groups increased rapidly during the first 6 h and then gradually leveled off from 6 to 18 h. After 18 h the concentration was significant higher (*p <* 0.05) in the US+Mx-LyzMBs group (134.98 ± 14.62 μg/cm^2^) and the US+LyzMBs+Mx group (85.88 ± 5.78 μg/cm^2^) than in the US+Mx group (68.79 ± 6.01 μg/cm^2^), Mx-LyzMBs group (56.68 ± 6.80 μg/cm^2^), Mx group (46.71 ± 9.23 μg/cm^2^), and LyzMBs group (13.87 ± 10.87 μg/cm^2^). The concentration did not differ significantly (*p* < 0.05) between the US+Mx and Mx-LyzMBs groups or between the Mx-LyzMBs and Mx groups. The penetration and deposition of Mx at 3 h in the US+Mx-LyzMBs group were 2.1 and 2.3 times higher than in the US+LyzMBs+Mx and US+Mx groups, respectively. Table 2 indicates that the amount of Mx deposited in the skin did not differ significantly among all of the groups after 18 h (*p* > 0.05). The total amount of Mx that penetrated was significantly greater in the US+Mx-LyzMBs group than in the other four groups.

**TABLE 2:** Amounts of Mx that after 18 h had permeated the skin, deposited on the skin, and penetrated across the skin. The concentration of Mx was significant higher (**p < 0.01) in the US+Mx-LyzMBs group and the US+LyzMBs+Mx group than in the US+Mx, Mx-LyzMBs, Mx, and LyzMBs groups. The concentration did not differ significantly between the US+Mx and Mx-LyzMBs groups or between the Mx-LyzMBs and Mx groups (*p* > 0.05). The amount of Mx deposited in the skin did not differ significantly among all of the groups after 18 h (*p* > 0.05). Data are mean±SEM values.

Parameter	Skin weight (g)	Deposited (μg/cm^2^)	Penetrated (μg/cm^2^)	Permeated (μg/cm^2^)
Group				
Mx	0.35 ± 0.13	25.69 ± 0.93	46.71 ± 9.23	72.39 ± 0.92
Mx-LyzMBs	0.33 ± 0.15	24.38 ± 2.67	56.68 ± 6.80	81.06 ± 2.67
US+Mx	0.33 ± 0.14	23.17 ± 0.99	68.79 ± 6.01	91.96 ± 0.99
US+LyzMBs+Mx	0.35 ± 0.21	24.89 ± 0.86	85.88 ± 5.78**	110.77 ± 0.86**
US+Mx-LyzMBs	0.34 ± 0.14	24.27 ± 2.63	134.98 ± 14.62**	159.25 ± 2.64**

Table 3 presents the Lyz concentrations in the three groups for percutaneous penetration and deposition over 18 h as analyzed using the ELISA reader. The total amount of Lyz was 3.5 times greater in LyzMBs group than in the control group, and the penetration and deposition of Lyz were 2.5 and 38.3 times higher in the US+LyzMBs group than in the control group.

**TABLE 3:** Amounts of Lyz that after 18 h had permeated the skin, deposited on the skin, and penetrated across the skin. The total amount, penetration and deposition of Lyz was greater in US+LyzMBs group than in other two groups (**p < 0.01). Data are mean±SEM values.

Parameter	Time (hours)	Skin weight (g)	Deposited (μU/mL)	Penetrated (μU/mL)	Permeated (μU/mL)
Group					
Control	18	0.08 ± 0.01	15.98 ± 0.33	3.10 ± 0.63	19.08 ± 0.96
LyzMBs	18	0.07 ± 0.01	9.64 ± 1.38	57.97 ± 5.00**	67.61 ± 6.39**
US+LyzMBs	18	0.09 ± 0.03	39.76 ± 4.37**	118.77 ± 10.30**	158.53 ± 14.67**

### Penetration Depths of FITC, Mx, and Lyz in Skin With US-Mediated LyzMB Cavitation

**FIGURE 6:**
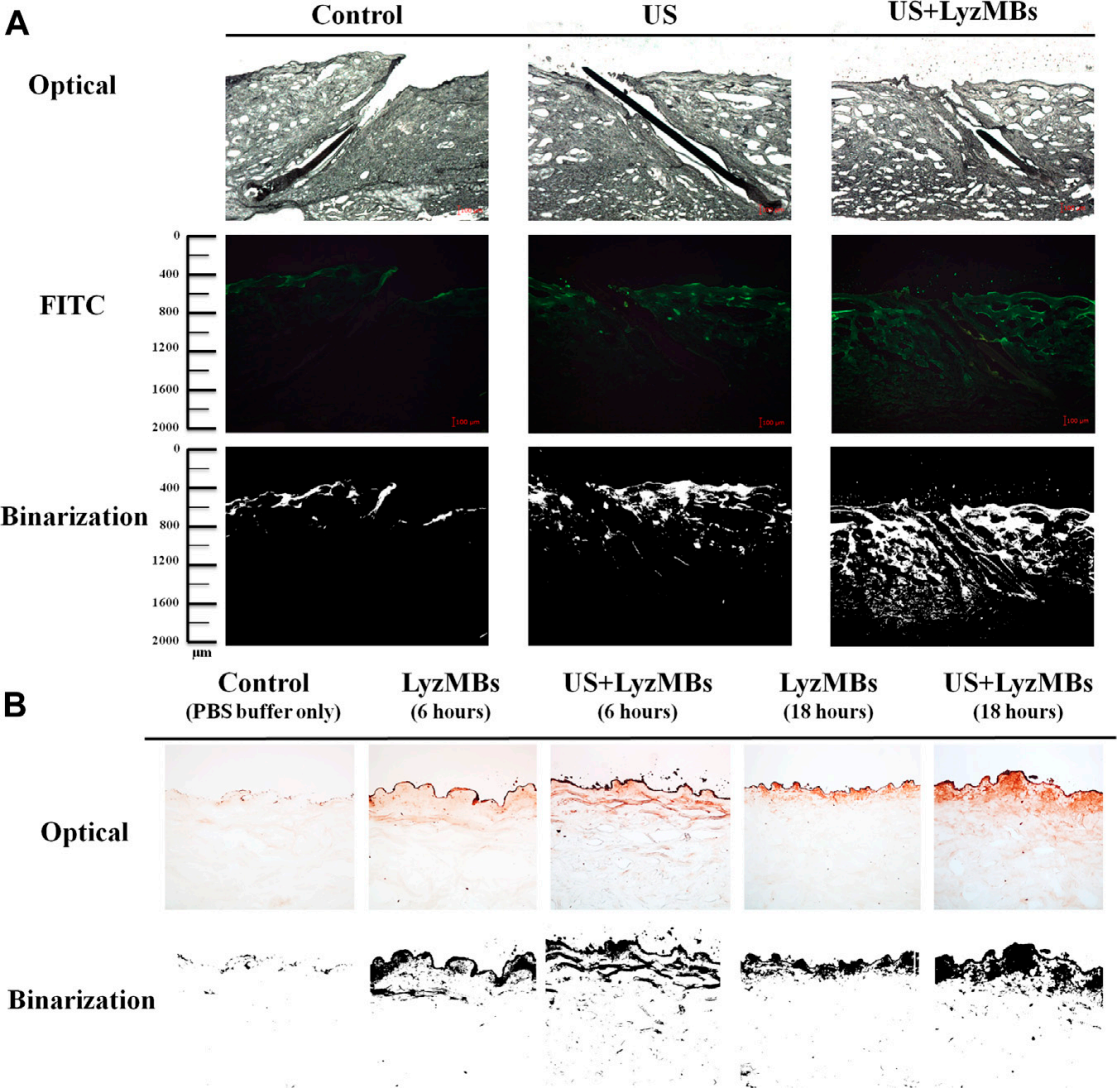
**(A)** *Bright*-*field* microscopy **(upper panels)**, fluorescence microscopy **(middle panels)**, and binarization **(lower panels)** images of the model drug FITC in the control, US, and US+LyzMBs groups after the FITC solution had been present for 6 h **(B)** Results of enzyme immunohistochemistry of Lyz in pigskin after 6 and 18 h in the control, LyzMBs, and US+LyzMBs groups.

As shown in Figure 6A, the *in vitro* skin permeation experiments demonstrated the ability of US-mediated LyzMB cavitation to act on the hair follicle of drugs relative to the US group. The fluorescence images were converted into grayscale images for quantitative calculations with image binarization (see lower panels of Figure 6A). The intensities of the fluorescence signals transmitted from the model drug FITC detected in the histology sections were approximately 3.6 and 2.0 times higher in the US+LyzMBs group (penetration depth = 1,267.3 ± 95.2 μm) than in the FITC-alone (control) group (penetration depth = 350.5 ± 34.5 μm) and the US group (penetration depth = 618.8 ± 79.6 μm), respectively, when the FITC solution was present for 6 h. The detected fluorescence signal was weaker for the control and US-treated skin sites, and identical results were obtained for all hair follicles in all tissue samples in the US+LyzMBs group. Figure 6B shows that enzyme immunohistochemistry detected more colored reaction products at 6 and 18 h in the US+LyzMBs group (6 h:11.8%, 18 h: 12.6%) than in the control (cannot be detected) and LyzMBs (6 h:7.6%, 18 h: 6.1%) groups.

### Growth of Murine Vibrissae Follicles

**FIGURE 7:**
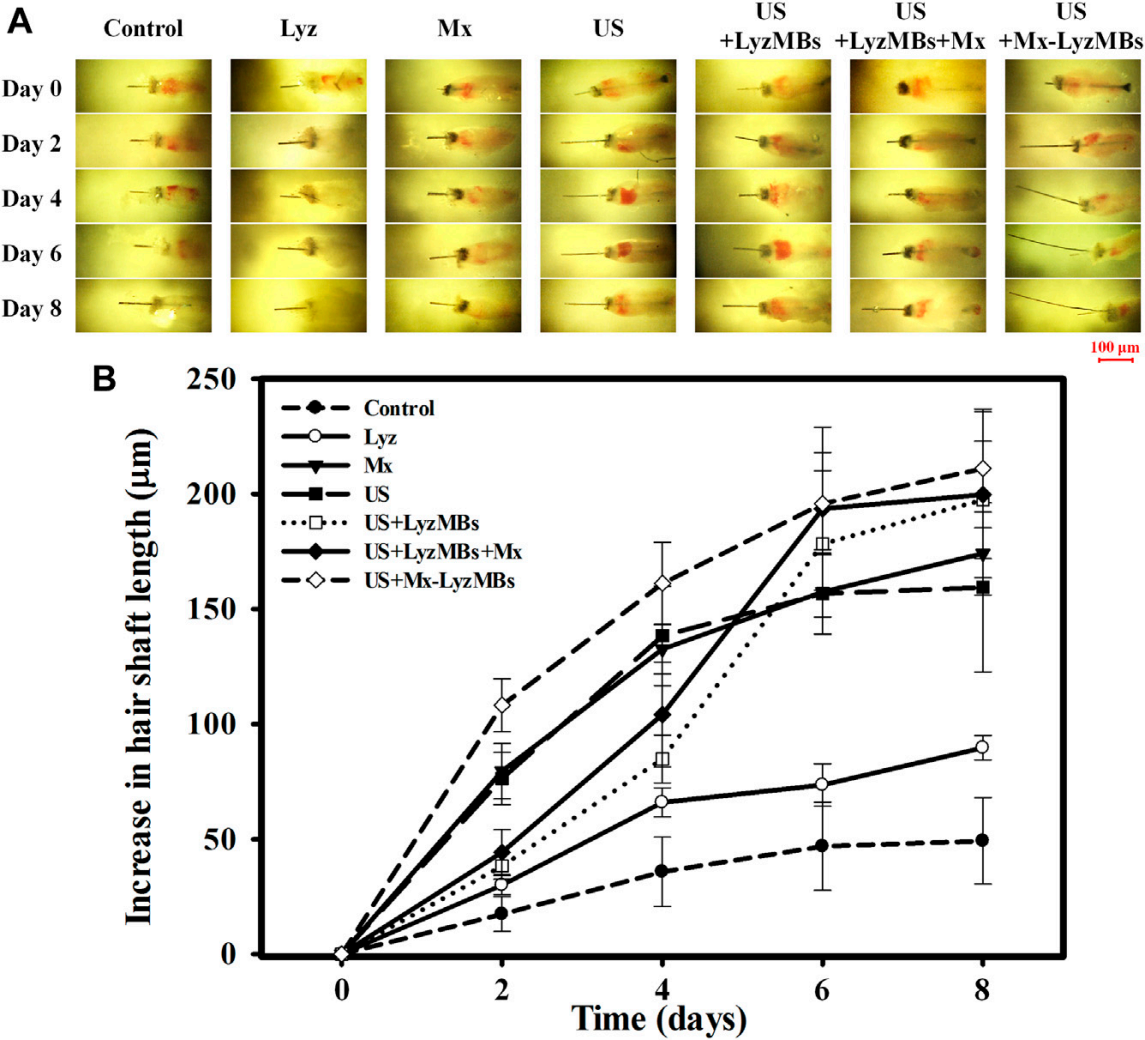
**(A)** Representative photographs of vibrissae cultured with or without treatments in an *in vitro* vibrissae culture system. **(B)** Growth curves for the vibrissae shafts over time in the seven treatment groups. Data are mean and SEM values.

Before applying the treatments, the optimal US exposure parameters for LyzMB destruction *in vitro* were investigated, which indicated that Mx-LyzMBs at the optimal concentration of 7 × 10^6^ MBs/ml were destroyed for 30 s of US exposure at a power density of 1 W/cm^2^ four times (Supplementary Figure S1). Consequently, US exposure for 30 s four times was used in the subsequent *in vitro* experiments. An organ culture model was used to examine the effects of Lyz, Mx, US, US+LyzMBs, US+LyzMBs+Mx, and US+Mx-LyzMBs on the regulation of vibrissae follicle hair growth. At day 2, the gross images (Figure 7A) and growth curves (Figure 7B) indicated that vibrissae were significantly longer in the US+Mx-LyzMBs group (108.18 ± 11.51 μm) than in the following groups: control (17.45 ± 7.55 μm) (*p <* 0.001), Lyz (30.07 ± 4.10 μm) (*p <* 0.001), Mx (79.55 ± 12.00 μm) (*p <* 0.01), US (76.39 ± 11.32 μm) (*p <* 0.01), US+LyzMBs (38.38 ± 5.78 μm) (*p <* 0.01), and US+LyzMBs+Mx (44.34 ± 9.79 μm) (*p <* 0.05). The trend of growth rate at day 4 was approximately the same as that at day 2 in all groups. At day 6, most treatment groups reached the plateau phase, with no significant difference in the length of vibrissae between the US+Mx-LyzMBs, US+LyzMBs+Mx, and US+LyzMBs groups. These data also show that both Mx and US, combined treatments of US and LyzMBs, and US combined with Mx and LyzMBs can promote vibrissae growth, respectively.

### *In Vivo* Hair Growth Enhancements in the US, US+Mx, US+LyzMBs, US+LyzMBs+Mx, and Mx-LyzMBs Groups

**FIGURE 8:**
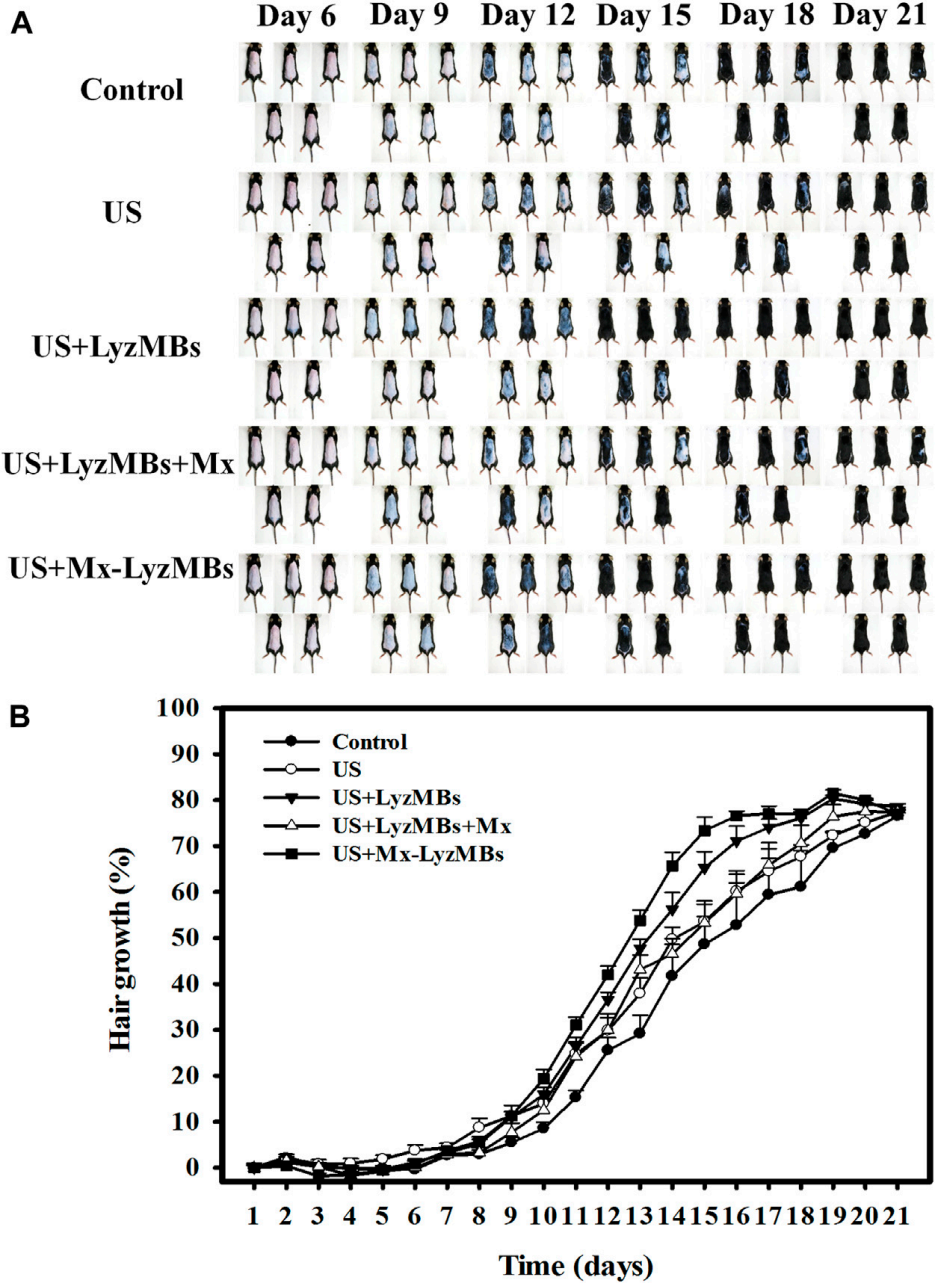
**(A)** Gross observations of the dorsal skin of C57BL/6 mice. The dorsal skin surfaces of the mice were shaved, and then test compounds and treatments were topically applied for 3 weeks. **(B)** Quantification of hair growth rates on the dorsal skin after shaving the hair in various mouse groups over 21 days. Data are mean and SEM values.

Figure 8A shows photographs of mouse skin in a completely untreated animal (day 1) and in the control, US, US+LyzMBs, US+LyzMBs+Mx, and US+Mx-LyzMBs groups at various time points after treatment. At day 9, the skin brightness for the five mice was lower in the US+Mx-LyzMBs group than in the following groups: US+LyzMBs+Mx (decreased in three mice), US+LyzMBs (decreased in four mice), US (decreased in three mice), and control (decreased in two mice). At day 12, the hair growth was greater in all mice in the US+Mx-LyzMBs group than in the other four groups. Figure 8B demonstrates the effects of Mx on dorsal hair growth over 21 days. At days 10 and 14, the growth rates in the US+Mx-LyzMBs group had increased by 19.4 and 65.7%, respectively. At day 14 there were obvious significant differences (*p* < 0.05) between the US+Mx-LyzMBs group (65.7%) and the other four groups (control = 41.7%, US = 49.7%, US+LyzMBs = 56.2%, US+LyzMBs+Mx = 46.6%). At day 16, the growth rate had reached a plateau in the US+Mx-LyzMBs group, with an increase of 76.6%, while the growth rates in the control, US, US+LyzMBs, and US+LyzMBs+Mx groups had increased by 52.8%, 60.2, 71.1%, and 59.7%, respectively. At that time point the growth rate did not differ significantly between the US+Mx-LyzMBs and US+LyzMBs groups (*p >* 0.05).

**FIGURE 9:**
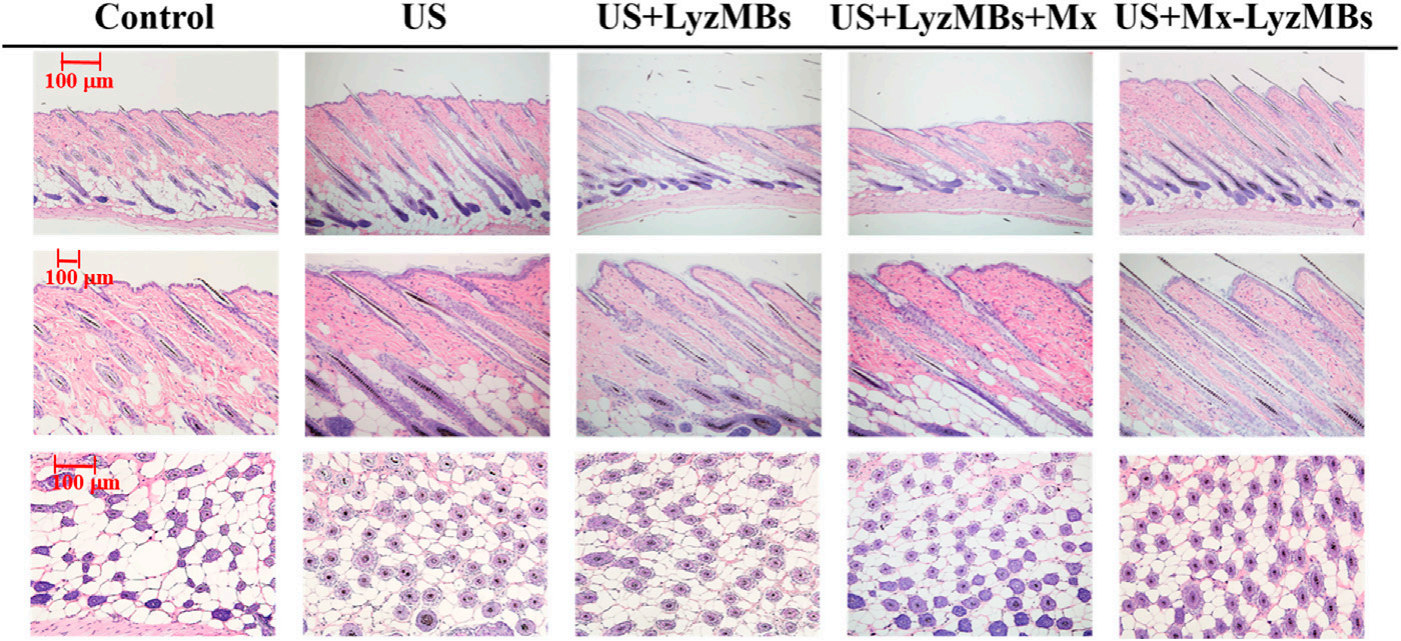
Histological observations of hair follicles in mice skin. The dorsal skin surfaces of the mice were shaved, and then test compounds and treatments were topically applied for 3 weeks. Vertical **(upper and middle panels)** and coronal **(lower panel)** views of the hair follicles.

The histology images in Figure 9 indicate that no skin damage was evident in any of the US treatment groups. Histological analyses of transverse sections (upper and middle panels of Figure 9 and Table 4) revealed significant increases in the diameter of the keratinized hair shaft after 21 days in the US, US+LyzMBs, US+LyzMBs+Mx, and US+Mx-LyzMBs groups, with the increases being largest in the US+Mx-LyzMBs and US+LyzMBs+Mx groups. Moreover, vertical sections revealed that the combination of US and Mx-LyzMBs promoted the elongation of hair follicles from the epidermis down to the subcutis. The histological analysis of coronal sections (lower panels of Figure 9 and Table 4) revealed that the numbers of hair follicles had increased after treatment. The diameters of the keratinized hair shafts and the numbers of hair follicles were both increased in the US+LyzMBs, US+LyzMBs+Mx, and US+Mx-LyzMBs groups, and especially in the latter two groups.

**TABLE 4:** Results of histological analyses of the numbers of hair follicles and the diameters of the keratinized hair shafts after 21 days of treatment. The diameters of the keratinized hair shafts and the numbers of hair follicles were both greater in the US+LyzMBs+Mx and US+Mx-LyzMBs groups than in other three groups (*p < 0.05).

	Control	US	US+LyzMBs	US+LyzMBs+Mx	US+Mx-LyzMBs
Follicle count	43.4 ± 4.50	46.6 ± 6.08	53.6 ± 6.08	58.8 ± 5.44*	62.2 ± 7.44*
Hair follicle diameter (μm)	34.9 ± 6.91	42.1 ± 9.52	42.8 ± 10.11	48.5 ± 9.43*	50.5 ± 14.87*

## Discussion

Combinations of Mx and topical antibacterial agents have been shown to improve hair growth (Kligman, 1991; Khandpour et al., 2002; Berger et al., 2003). A previous study found that Lyz can stimulate mouse hair follicle growth *in vitro* (Su et al., 2015). Lyz is a potent bactericidal agent against both Gram-negative and Gram-positive bacteria regardless of its enzyme activity. The antimicrobial activity of Lyz remains after LyzMBs have been formed (Cavalieri et al., 2008). The present study therefore combined Mx-LyzMBs with US to enhance the efficacy of Mx for hair growth treatment. Our previous study created layer-by-layer Mx-coated albumin-shelled MBs and combined them with sonication by US energy in the water phase to enhance hair growth (Liao et al., 2016b). The maximum loading efficiency of Mx on the Mx-COL-MBs (with COL:Mx = 1:1, COL: chitosan oligosaccharide lactate) was only 14.87%. In the present study, the encapsulation of Mx coated on LyzMB shells achieved a loading efficiency of Mx-LyzMBs of 20.33 ± 2.37%. Mx was grafted directly as the first layer on LyzMBs, which resulted in higher loading efficiency than for albumin-shelled MBs.

Our *in vitro* experiments revealed that the efficiency of releasing Mx from LyzMBs was higher at a high pH value (pH 7.4), while the application of US energy could enhance drug release. The activity of Lyz was most stable at pH 5.2, and its activity was significantly affected at higher temperatures and pH (Venkataramani et al., 2013). This might be due to the activity of Lyz being more unstable at pH 7.4 than at pH 5, and leads to an unstable conjugation between Mx and LyzMBs at pH 7.4 that increases the amount of Mx released from LyzMBs. The release efficiency of Mx-LyzMBs differs from that of Mx-COL-MBs, whose release efficiency is higher at a low pH value (pH 4) (Liao et al., 2016b). Exposing the skin to commercial cleansing products (whose *pH* values are typically between 6 and 8.5) results in the skin pH increasing up to 6 h after application (Lambers et al., 2006). Although the pH value of the scalp (like the rest of the skin) is around 5.5 (Gavazzoni Dias et al., 2014), the Mx released from LyzMBs at pH 7.4 reached the plateau after 6 h in the present study. Therefore, the natural pH value of the scalp did not affect the amount of Mx released from LyzMBs within 6 h.

Our previously revealed *in vitro* permeation profiles of Mx through skin samples indicate that the concentrations in the US+MBs+Mx group increased more rapidly relative to the other groups during the first 6 h, and then became closer to or even lower than that in the US+Mx-COL-MBs group from 6 to 18 h (Liao et al., 2016b). The present results indicate that combining US with Mx-LyzMBs increased the permeation more rapidly relative to the other groups at all measurement time points. The current MBs-based drug carrier system of Mx was developed using LyzMBs. Moreover, the results for percutaneous penetration and deposition over 18 h showed that the total amount of Lyz increased more in the LyzMBs and US+LyzMBs groups than in the control group. Therefore, LyzMBs affects the amounts of Lyz deposited on the skin and penetrated across the skin at 18 h.

In experiments of the FITC penetration depth in pigskin, combining US with LyzMBs resulted in more efficient and deeper penetration into the hair follicles compared to the US group, and in the enzyme immunohistochemistry experiments there were more chicken Lyz antigens detected in US+LyzMBs groups than in LyzMBs alone after 6 and 18 h of penetration. These findings indicate that US can enhance the delivery of LyzMB fragments closer to the target structure of interest in the hair follicles.

The murine vibrissae culture experiments demonstrated that the vibrissae growth was greater in the US+Mx-LyzMBs group than in the other groups, and more stable than that in the US+LyzMBs+Mx group on all of the observation days. These findings indicate that Lyz alone can promote mouse vibrissae growth, which is consistent with the findings of a previous study (Su et al., 2015). In our C57BL/6 mice hair growth experiments, the hair growth rates from day 14–16 increased more significantly in the US+LyzMBs group than in the other groups, with the exception of the US+Mx-LyzMBs group. The results support that combining US and LyzMBs can promote hair growth. Compared to LyzMBs+Mx, the hair growth rates were more stable when Mx was grafted on the LyzMBs. Moreover, the histological experiments also indicated that the diameters of the keratinized hair shafts and the numbers of hair follicles were significantly increased in the US+Mx-LyzMBs group compared to in the other groups.

## Conclusion

This study has yielded new findings for combining LyzMBs with US to promote Mx to enhance hair growth. Mx can be grafted directly as a layer on LyzMBs, which results in a higher loading efficiency than for albumin-shelled MBs. US can enhance the delivery of LyzMB fragments closer to the target structure of interest in the hair follicles. Therefore, LyzMBs affect the amount of Lyz that is deposited on the skin or penetrates across the skin. Combining US with Mx-LyzMBs was found to increase Mx release more rapidly relative to the LyzMBs+Mx group at all measurement time points.

These findings indicate that a MBs-based drug carrier system for Mx could be developed using LyzMBs. Most importantly, US combined with Mx-LyzMBs can significantly enhance hair growth when Mx is grafted on the LyzMBs.

## Data Availability

The original contributions presented in the study are included in the article/Supplementary Material, further inquiries can be directed to the corresponding authors.
