# Supplementary figures and images for: Minoxidil-Coated Lysozyme-Shelled Microbubbes Combined With Ultrasound for the Enhancement of Hair Follicle Growth: Efficacy In Vitro and In Vivo
Source: Front Pharmacol. 2021 Apr 27;12:668754. doi: 10.3389/fphar.2021.668754 (PMC8111400; doi:10.3389/fphar.2021.668754)

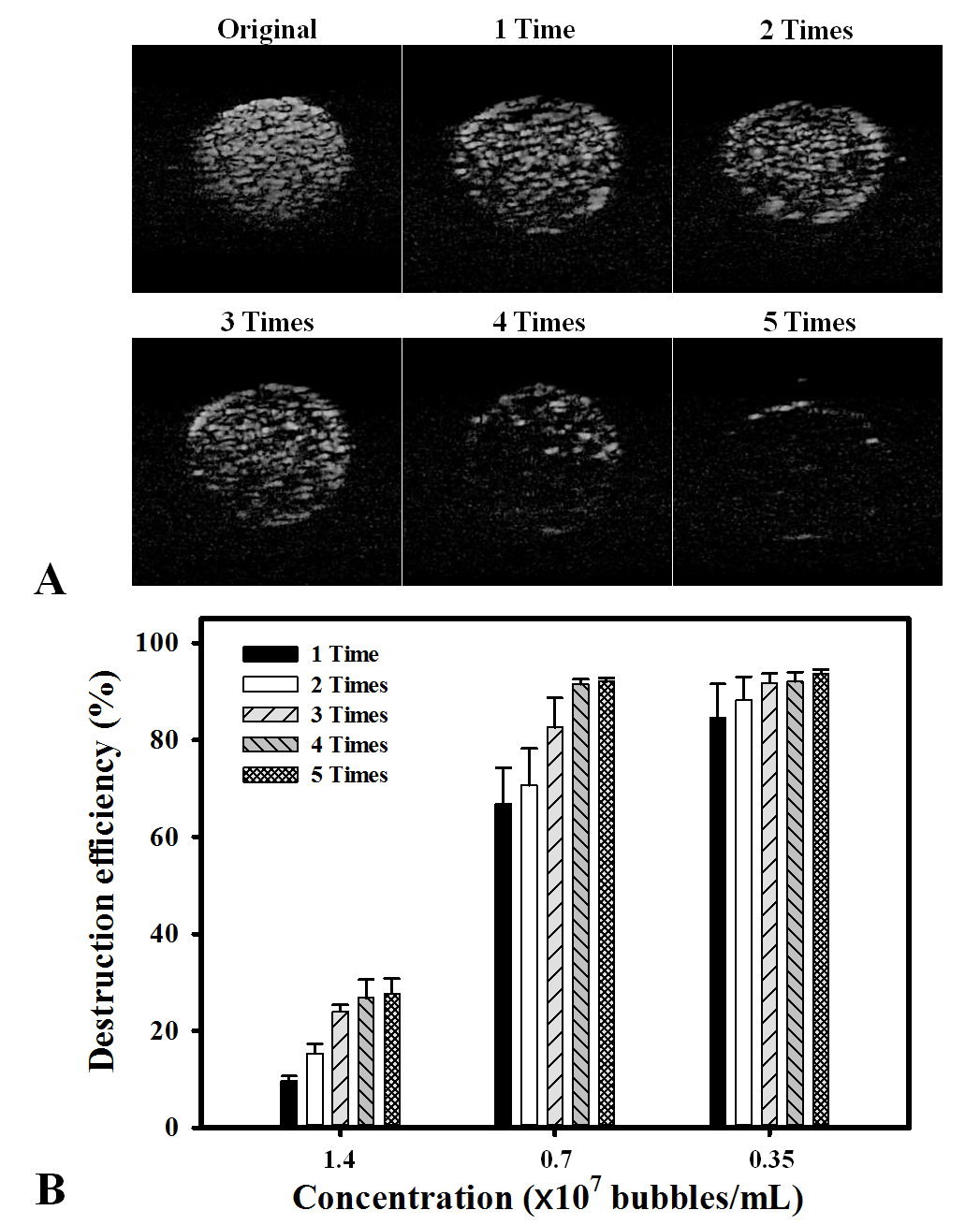

Supplement: Supplementary file 1 [file image1.tif]
