# Supplementary material for: Minoxidil-Coated Lysozyme-Shelled Microbubbes Combined With Ultrasound for the Enhancement of Hair Follicle Growth: Efficacy In Vitro and In Vivo
Source: Front Pharmacol. 2021 Apr 27;12:668754. doi: 10.3389/fphar.2021.668754 (PMC8111400; doi:10.3389/fphar.2021.668754)
Supplement: Supplementary file 2 [file datasheet1.docx]

Supplemental Results


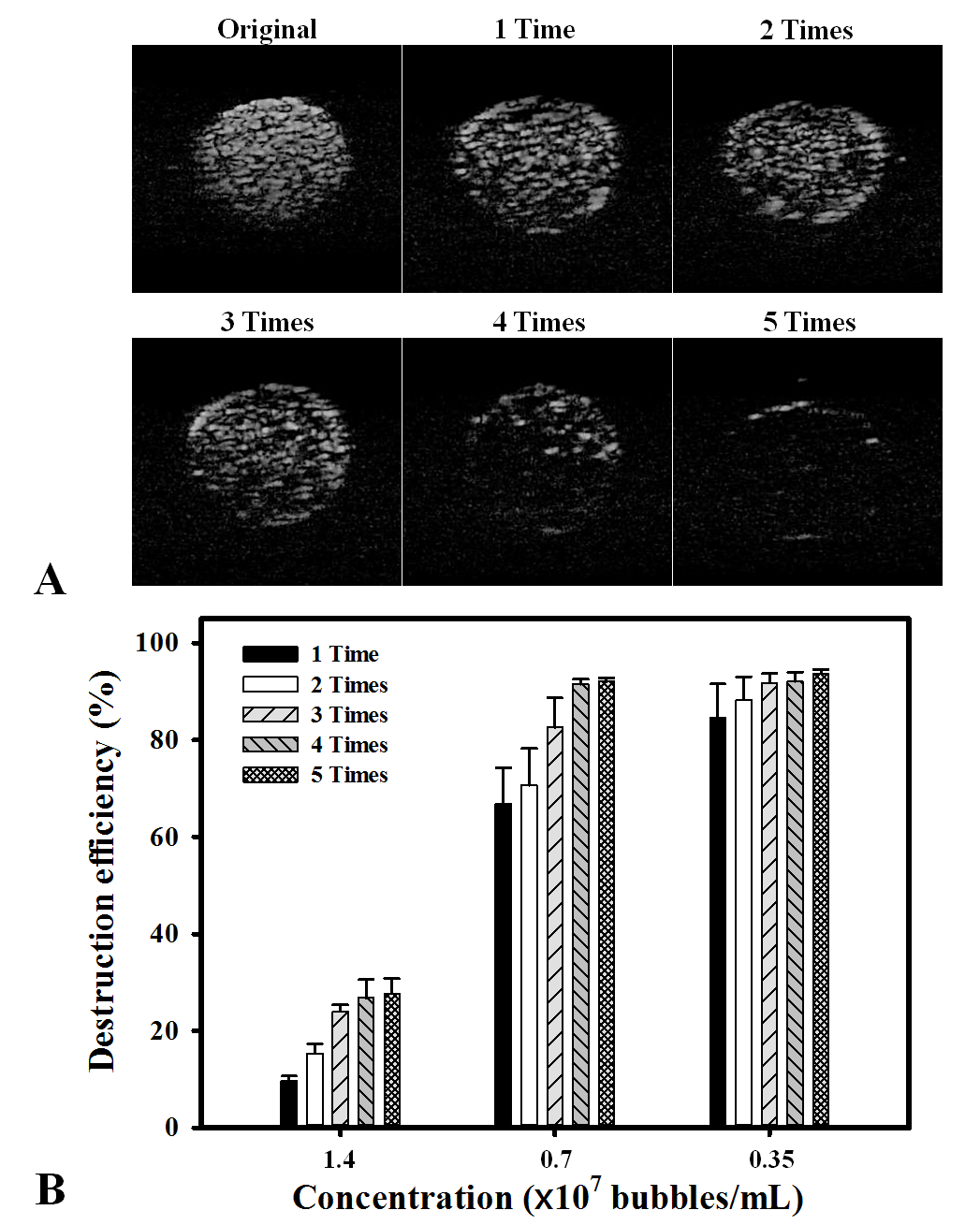


Figure 1. High-frequency US images of MBs without and with US exposure. (A) Images for US power exposure of 1, 2, 3, 4, and 5 times for 30 seconds/times, respectively. (B) Quantification of MBs destructions (*n*=5). Data are mean and SEM values.
